# Relationship between Intraoperative Bile Culture Outcomes and Subsequent Postoperative Infectious Complications: A Retrospective Cohort Study

**DOI:** 10.1155/2024/3930130

**Published:** 2024-05-20

**Authors:** Jaime Alberto Ramírez-Arbeláez, Ricardo Leonel Arroyave-Zuluaga, Luis Manuel Barrera-Lozano, Verónica Hurtado, Daniel González-Arroyave, Carlos M. Ardila

**Affiliations:** ^1^Department of Transplants, Hospital San Vicente Fundación, Rionegro, Colombia; ^2^Department of General Surgery, Universidad de Antioquia UdeA, Medellín, Colombia; ^3^Department of Surgery, Universidad Pontificia Bolivariana, Medellín, Colombia; ^4^Universidad de Antioquia UdeA, Medellín, Colombia

## Abstract

The presence of positive bile culture during intraoperative procedures has been associated with elevated morbidity and mortality rates in hepatobiliopancreatic surgeries, contributing to increased healthcare expenditures. However, the precise impact of bactobilia on the development of postoperative complications remains uncertain due to existing disparities in the published literature. In this retrospective cohort study, we assessed 137 patients who underwent major hepatobiliopancreatic surgery to examine the relationship between intraoperative bile culture outcomes and subsequent postoperative infectious complications. Among patients with bactobilia, a significant 35.1% exhibited systemic or local infectious complications, whereas only 11.1% of those with negative culture results experienced any infectious complications (*p* = 0.002). Similarly, a notable difference was observed in the incidence of surgical site infections, with 24.3% in the bactobilia group compared to 7.9% in the negative culture group (*p* = 0.01). A total of 74 monomicrobial cultures with microbiological growth were isolated, predominantly featuring Gram-negative microorganisms, primarily *Enterobacteriaceae* in 49 cultures. *Escherichia coli* was identified in 37.8% of positive cultures, while *Klebsiella pneumoniae* was evident in 21.6%. Gram-positive microorganisms were present in 10 cultures, with *Enterococcus* emerging as the prevailing species. The logistic regression model identified a positive bile culture as an independent factor significantly associated with infection development (OR: 2.26; 95% confidence interval: 1.23-11; *p* = 0.02). Considering the limitations of the study, these findings underscore the critical importance of conducting bile cultures during the intraoperative phase to enable vigilant monitoring and prompt management of infectious complications.

## 1. Introduction

The bile duct, crucial for digestive and excretory processes, transports bile from the liver to the small intestine, emphasizing its clinical significance [1]. Maintaining its sterility, attributed to the sphincter of Oddi and bile salts' bacteriostatic properties, is vital, with endotoxins acting as a barrier against bacterial translocation [1, 2]. This sterile environment is essential for medical interventions and physiological balance, playing a pivotal role in health preservation and complication prevention [1].

Bactobilia, the presence of bacteria in sterile bile ducts and gallbladder, leads to various clinical implications, including localized infections, cholangitis, and broader complications such as biliary colic and postoperative issues [2, 3]. Timely detection and effective management of bactobilia are crucial to mitigate its adverse effects on health [2, 3]. Factors such as medical procedures, gallstones, and bile duct obstructions contribute to bacterial presence in bile [2]. Additionally, risk factors like advanced age, male gender, and coronary artery disease are associated with bactobilia [3]. Hospital-acquired infections, particularly from surgeries, are significant burdens, both financially and in terms of lives lost, with surgical site infections ranking second among nosocomial infections [4, 5].

In hepatobiliary disease, bactobilia can result from various causes, with postoperative infectious morbidity around 27% [6]. Morbidity rates in hepatobiliary and pancreatic surgery vary from 30% to 60%, with mortality rates of 3% to 5% [7]. Perioperative infections, including operative site infections, are major contributors to this morbidity [8, 9]. Preoperative and intraoperative factors such as cardiovascular disease, diabetes, obesity, malnutrition, and surgical variables contribute to perioperative complications [7, 10, 11].

Previous studies emphasize the effectiveness of advanced surgical techniques, innovative suturing methods, perioperative conditioning, and surveillance protocols in reducing perioperative morbidity [10, 11]. However, the relationship between bactobilia and surgical infections varies across studies [12, 13]. Some studies show a correlation between postoperative infections and positive cultures, while others find no discernible difference [14, 15]. Recent cohort studies suggest that patients with positive bile cultures have higher rates of isolated infections when exposed to perioperative prophylaxis compared to extended prophylaxis [16]. Conversely, isolated infections unrelated to positive bile cultures have been reported [17].

Intraoperative bile cultures, investigated in two studies, do not provide additional insights into postoperative infectious complications [9, 18]. Notably, there is inconsistency between bile cultures and cultures from infection sites, raising concerns about tailoring treatment based on intraoperative bile cultures [19, 20]. Routine implementation of bile cultures may need reconsideration due to the infrequency of isolated infections and similar probabilities of abdominal infectious complications between patients with positive and negative bile cultures [17].

Considering the preceding concerns, this study is aimed at investigating the outcomes of intraoperative bile culture and its potential connection with subsequent postoperative infectious complications in patients undergoing hepatobiliopancreatic surgical procedures. This study hypothesizes that patients who have a positive bile culture are more likely to develop an infection after hepatobiliopancreatic surgery.

## 2. Materials and Methods

### 2.1. Study Design and Participants

The study examined individuals aged 18 and above who underwent hepatobiliopancreatic surgery at Hospital San Vicente Fundación in Rionegro, Colombia. The study cohort includes patients with bile cultures collected during surgery from November 2018 to December 2022. Patients who lacked recorded bile culture were excluded from this analysis.

### 2.2. Data Collection

Clinical and demographic variables were retrospectively compiled from the patients' medical records. Data compilation was performed independently by two authors, with a third impartial investigator involved to resolve any discrepancies.

The significant variables included patient characteristics, cardiovascular risk profiles, relevant preoperative factors, types of surgical interventions, postoperative complications, duration of hospitalization, and identification of microorganisms in bile samples. A thorough review of clinical variables and laboratory assessments was conducted, including comprehensive analysis of bacterial typing and culture outcomes. Postoperative surgical complications were classified according to the well-established Dindo-Clavien classification [21].

### 2.3. Microbiological Procedures

The procedure for bile sample collection was executed as follows: Following the incision of the bile duct, a practice observed during both reconstruction procedures and interventions such as Whipple surgery or hepatectomies, a minimum of 2 ml of bile was promptly extracted and placed into a sterile dry receptacle. The sample was then expeditiously conveyed to the institutional microbiology laboratory. Conventional microbiological procedures were conducted to identify the organisms [22]. Subsequent processing transpired within a biosafety chamber, whereby the sample was aseptically introduced into culture media using a sterile pipette. This entailed depositing two or three droplets directly onto the culture media, encompassing blood agar, MacConkey agar, and thioglycolate or brain heart infusion. Inoculation transpired via exhaustion in an inverted G configuration, with all media subsequently subjected to incubation at a controlled temperature of 35.5°C ± 2 for a duration of 48 hours. Noteworthy is the utilization of an enriched CO_2_ atmosphere (5%) for the incubation of blood agar. The identification of bacterial strains was predicated on the inception of a microbial suspension into specialized cards, each equipped with distinct panels of biochemical reactions. The ensuing interpretation was facilitated by an advanced colorimetric optical system, capable of assessing trichromatic wavelengths. Bile samples were considered sterile if there was no visible growth within a 48-hour period. The patients were categorized into two cohorts according to their bile culture outcomes, and subsequently, their postoperative progress and mortality rates were compared in a retrospective manner.

### 2.4. Sample Size Calculation

According to the literature, there is a general incidence of infection of 10% (p1), which increases to 40% (p2) when a positive bile culture is detected. For a two-sample comparison of percentages, the estimated sample size should be at least 32 patients for each subgroup (patients with positive bile culture versus those with negative culture status). This estimation assumes an alpha of 5%, a power of 80%, and equal sample sizes [23].

### 2.5. Statistical Analysis

Statistical analysis was performed using R version 3.3.5 software with the Excel package. Normal distribution of data was assessed using the Kolmogorov-Smirnov test. Descriptive analysis included relative frequencies. Continuous variables were compared using Student's *t*-tests and summarized using means and standard deviations. Categorical variables were compared using chi-square tests, with frequencies and percentages presented. Significance was determined for *p* values less than 0.05.

For multivariate analysis of surgical site infection, a logistic regression model was constructed and adjusted for relevant confounding variables. The adequacy of the logistic model was evaluated using the Hosmer-Lemeshow test. Interaction effects were examined using ANOVA to identify differences between the model with the interaction variable and the model without it.

### 2.6. Bioethical Considerations

The protocol of this study was approved by the Institutional Bioethics Committee of Hospital San Vicente Fundación (06-2023). Furthermore, the execution of this investigation strictly adhered to all parameters outlined by the Declaration of Helsinki.

## 3. Results

**Figure 1:**
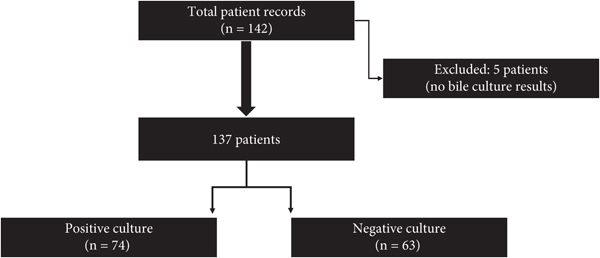
Flowchart for selecting study participants.

A total of 142 patient records of individuals who underwent surgical procedures were procured. After the exclusion of 5 patients who did not furnish bile culture results, the resulting study cohorts comprised 63 patients with negative culture outcomes and 74 patients exhibiting positive culture results. Accordingly, the analysis encompassed 137 patients who underwent surgical intervention, with intraoperative bile culture processing conducted during the specified study assessment period (Figure 1).

### 3.1. General Characteristics, Cardiovascular Risk Factors, and Preoperative Factors

Table 1 illustrates the absence of statistically significant disparities in both age and gender distribution among patients within the two study groups. Similarly, no notable statistical differences were detected in certain cardiovascular risk factors. Comparable findings emerged concerning other preoperative variables, encompassing prealbumin levels before the procedure, as well as intraoperative aspects such as bleeding surpassing 400 ml and the requirement for intraprocedural transfusions, with no significant differences evident between the groups. Nonetheless, discernible statistical significance was noted pertaining to preoperative biliary drainage, which displayed heightened frequency in the cohort with positive cultures. Furthermore, a marked diminution in serum albumin levels was noticeably evident within the same group of patients.

### 3.2. Surgical Procedures

Among the entirety of surgical interventions, a total of 66 cases encompassed Whipple surgery (proximal pancreaticoduodenectomy), 41 cases involved bile duct reconstructions (hepaticojejunostomy), and 30 cases constituted hepatectomies, involving some form of manipulation of the bile duct. No noteworthy disparities surfaced between the two groups (positive bile culture versus negative bile culture) concerning Whipple surgery and hepatectomies. However, an evident difference emerged in the context of bile duct reconstructions, where a pronounced and statistically significant elevated mean of positive cultures was observed (37.8% vs 20.6%) (Table 2).

### 3.3. Postoperative Complications, Duration of Hospitalization, and Mortality

Both infectious and noninfectious general complications manifested in 45% of the study population; nevertheless, this proportion was notably higher within the culture-positive subgroup (52% versus 36%), although without statistical significance. Among patients exhibiting bactobilia, 35% encountered systemic infectious complications such as pneumonia, urinary tract infections, bacteremia, or local complications encompassing operative site infections, cholangitis, or fistulas. In contrast, merely 11% of patients displaying negative culture results experienced any form of infectious complication, revealing a statistically significant disparity (*p* < 0.05). A parallel trend was evident in surgical site infections, wherein substantial and statistically significant differences emerged (24% within the bactobilia group versus 7% among culture-negative patients).

Major complications, classified as grade > II according to established criteria [21], occurred in 17% of culture-positive patients compared to 11% of their counterparts with negative culture results, although statistical significance was not attained (*p* > 0.05).

Mortality was documented in 4% of patients exhibiting positive culture outcomes (6 individuals) and 8% (3 individuals) within the negative culture group (*p* > 0.05). Concurrently, the duration of hospitalization was reported as 10 days for the culture-positive group, and 8 days for the negative culture group (*p* > 0.05) (Table 3).

### 3.4. Microorganisms Isolated in Bile

A total of 74 monomicrobial cultures showcasing microbiological growth were successfully isolated, with Gram-negative microorganisms representing the predominant majority, primarily attributed to the presence of *Enterobacteriaceae* in 49 of the cultures. Among the positive cultures, *E. coli* was identified in 37.8% of cases, whereas *K. pneumoniae* was evident in 21.6%. Gram-positive microorganisms were discernible in 10 cultures, with *Enterococcus* emerging as the prevailing species. *Candida* growth was detected in a solitary culture instance (Table 4).

### 3.5. Multivariate Logistic Regression Analysis

To explore the impact of a positive culture on the occurrence of surgical site infections, a multivariate analysis was conducted employing an adjusted logistic regression model. The objective of the logistic regression model was to conduct multivariable analysis to understand how the various studied factors could predict the development of postoperative infection. This model incorporated variables deemed potentially influential as regressors. As depicted in Table 5, the sole variable demonstrating a significant capacity to elucidate the presence of surgical site infections was a positive culture (OR 2.26, 95% CI 1.23-11). Notably, other variables such as bleeding exceeding 400 ml, diabetes mellitus, age, and prealbumin levels less than 15 mg dl failed to attain statistical significance within the model.

The definition of surgical site infection was based on the classification provided by the Centers for Disease Control and Prevention (CDC) of the United States (Surgical Site Infection Event-SSI, National Healthcare Safety Network) and diagnosed through culture of secretion obtained from the wound or intra-abdominal collection [24]. Superficial infections limited to the skin and subcutaneous tissue were included. Deep infections encompassed fascia and muscles, as well as organ-space infection.

Age was assessed as a plausible confounder. Through residual-based comparisons, insufficient evidence emerged to substantiate the superiority of the model with age inclusion over its absence. Furthermore, the beta coefficient of the positive culture variable exhibited a disparity of less than 10 percent, substantiating the conclusion that age did not constitute a confounding factor.

An evaluation of interaction was executed by means of ANOVA, scrutinizing the model with and without the interaction variable (diabetes). With a resultant ANOVA *p* value of 0.19, it was deduced that compelling evidence was lacking to establish an interaction between diabetes and the presence of surgical site infections.

To gauge the suitability of the logistic model, the Hosmer-Lemeshow test was invoked. The test yielded a *p* value of 0.91, thereby providing ample grounds to reject the null hypothesis. This substantiated that the model was aptly aligned with the data, signifying its robust fit and explanatory capability.

## 4. Discussion

Microbiological identification of bile isolates is crucial for targeted treatment, infection management, and surgical decision-making in hepatobiliary infections. Its impact extends to clinical practice, research, and public health strategies, enhancing patient care and outcomes [20]. Collecting data on microbial isolates aids in epidemiological surveillance, identifying bacterial prevalence trends, antimicrobial resistance patterns, and emerging pathogens [14, 15, 20]. This information is essential for public health strategies and infection control measures. Additionally, microbiological identification guides timely management of early nosocomial infections, influencing hospitalization duration and mortality rates [20].

Bactobilia can involve a variety of microorganisms, both aerobic and anaerobic. The main microorganisms related to bactobilia include *Escherichia coli*, *Klebsiella pneumoniae*, *Enterococcus* species, *Streptococcus* species, *Pseudomonas aeruginosa*, *Staphylococcus aureus*, *Bacteroides* and *Clostridium* species, *Enterobacter* species, and *Citrobacter* species. It is important to note that the microbial profile of bactobilia can vary from one individual to another and may be influenced by factors such as underlying health conditions, recent medical procedures, and local microbial patterns. Microbiological identification of the specific organisms involved is crucial for accurate diagnosis and targeted treatment [14, 15, 20].

The significance of bactobilia in isolation, without concurrent data on other infections, remains a subject of ongoing debate. While some studies suggest a potential correlation between bactobilia and subsequent infections or complications, findings vary across published studies [2, 14–19]. Consequently, a consensus has yet to be established, resulting in a lack of compelling evidence supporting the universal recommendation for routine bile culture collection in all biliary procedures.

Hepatobiliopancreatic surgery encompasses a wide array of procedures aimed at treating disorders of the liver, bile ducts, and pancreas, ranging from benign to malignant conditions [9, 25]. Despite significant advancements in surgical techniques and perioperative care, this field remains inherently complex and challenging [26, 27]. As such, achieving favorable outcomes requires thorough evaluation and a multidisciplinary approach to treatment.

Hepatobiliary and pancreatic surgery continues to be associated with a substantial incidence of complications, ranging from 30% to 50%. Among these, infectious morbidity holds significant prominence and serves as a notable indicator of patient care quality [26, 27].

The potential for biliary contamination during surgical procedures may increase the incidence of postoperative infectious complications. Consequently, the current study is aimed at exploring the contributing factors associated with intraoperative bactobilia in hepatobiliary pancreatic surgery. A multivariate analysis was conducted to mitigate the influence of potentially confounding variables that could introduce bias to the observed effect of a positive culture on the manifestation of surgical site infections. Additionally, this investigation considered aspects such as bile duct reconstruction and the frequency of perioperative complications within the initial 30 days following surgery [26, 27].

Within the context of this present study, most complications were evident within the cohort of patients exhibiting microbial growth in their bile cultures. Notably, the bactobilia group featured a marked prominence of infectious complications, encompassing both local and systemic infections. However, due to the conflicting outcomes presented in published reports concerning the impact of preoperative internal biliary drainage alone on subsequent postoperative factors linked to its presence, such as bile contamination, retrospective investigations have been conducted. These studies involve the comparison of patient groups with and without colonized bile. Positive bile cultures were documented in 94% of patients who underwent preoperative internal biliary drainage, in contrast to 34% of patients who did not undergo such drainage [28]. However, no definitive association was established between infected bile and subsequent postoperative infectious or noninfectious complications and mortality. It is important to note that these conclusions might have been influenced by the inclusion of palliative bypass patients alongside those who underwent resectional procedures [15].

In a separate study, positive bile cultures were identified in 58% of patients who underwent preoperative biliary drainage. Multivariate analysis indicated a substantial correlation between preoperative biliary drainage and postoperative infectious morbidity and mortality [29]. The research also revealed that 47% of patients who underwent preoperative internal biliary drainage exhibited a positive bile culture, compared to 31% of those who did not receive such drainage. While preoperative internal biliary drainage was not directly linked to elevated rates of morbidity and mortality, a positive bile culture showed significant associations with overall morbidity, infectious morbidity, and mortality, albeit not with noninfectious morbidity following pancreaticoduodenectomy [30].

Another investigation indicated that positive bile cultures were detected in 80% of patients who underwent preoperative internal biliary drainage, compared to 14% of patients who did not undergo drainage. This suggests that a positive bile culture corresponds to higher overall and infectious morbidity rates [14]. It has been suggested that preoperative biliary drainage predisposes individuals to a favorable environment for a positive intraoperative bile culture. Patients exhibiting a positive intraoperative bile culture are notably subjected to a clinically significant heightened susceptibility to the development of infectious complications, as well as postoperative wound infections after pancreatic surgery [2]. Another study demonstrated that there were no statistically significant distinctions in postoperative infectious and noninfectious morbidity, or mortality, between groups of patients undergoing resectional procedures for periampullary malignancies, categorized as colonized and sterile based on bile culture results. While internal biliary drainage does introduce microorganisms into the biliary network, this colonization appears to have minimal impact on the susceptibility to infectious or noninfectious complications, as well as postoperative mortality [15]. Nevertheless, the outcomes of this investigation align harmoniously with prior research findings that underscore the criticality of prompt identification of infectious complications in surgeries involving bile duct reconstruction [2, 14, 28–31]. This identification is essential for guiding treatment strategies aligned with the specific isolates from intraoperative cultures [2, 14, 31].

Although all these studies are retrospective cohorts, interestingly, a prospective randomized clinical trial presented evidence that the administration of prophylactic antibiotics targeted based on preoperative bile culture findings resulted in a reduction of surgical site infections after hepatobiliopancreatic surgery [31]. In a prospective study, it was also observed that following bile duct stenting, 98% of patients exhibited positive bile cultures, whereas merely 21% of infected bile was detected in patients who did not undergo drainage (*p* < 0.001) [26]. Indeed, it has been suggested that the genesis of postoperative complications after hepatobiliopancreatic surgery appears to be a multifaceted phenomenon, and the colonization of bile could potentially be one of the contributory elements [15].

Within the examined population, the overarching prevalence of bactobilia stood at 54%. These observations exhibit alignment with reports pertaining to pancreaticoduodenectomy, hepatectomies, and bile duct reconstruction, where the prevalence ranges between 51% and 78%. Notably, in the context of bile duct reconstruction, a heightened correlation with positive culture outcomes is discernible, primarily due to the predominant inclusion of patients necessitating surgical intervention for bile duct injury [9, 12, 13].

In the case of pancreaticoduodenectomies, similar findings to those of this research have been observed in observational studies [32–34], systematic reviews, and meta-analyses [34, 35].

Mohan et al. noted an association between bactobilia and the onset of surgical site infections, suggesting its potential role as a guide for antibiotic selection [32]. Parapini et al. pointed out that positive bile cultures were linked to a greater occurrence of major complications and surgical site infections. Patients with sterile bile cultures exhibited the lowest risk of postoperative complications, suggesting that measures to reduce bacterobilia rates, such as limiting biliary instrumentation, should be taken into consideration [33]. Itoyama et al. noted that intraperitoneal contamination on postoperative day 3, featuring bacterial species like those found in intraoperative bile, showed a correlation with postoperative complications. They indicated that antibiotic sensitivity profile of these bacteria may aid in selecting optimal antibiotics to address infectious postoperative complications in pancreaticoduodenectomy [34].

The systematic reviews and meta-analysis indicated an association between a positive intraoperative bile culture and the likelihood of a patient developing a surgical site infection [9, 35]. Müssle et al. identified bacterobilia in nearly half of pancreaticoduodenectomies, and this was linked to a higher incidence of surgical site infections. The microbiome in intraoperative bile and postoperative infectious sources exhibited a concordance of approximately 50% in patients, offering the possibility of promptly administering targeted antibiotics and assessing resistance patterns [9].

Preoperative drainage of the bile duct prior to surgery has exhibited a substantial link with an elevated likelihood of bactobilia, often yielding notable figures ranging from 86% to 98%. In patient cohorts subjected to procedures such as endoscopic retrograde cholangiopancreatography, transparietohepatic cholangiography, or preceding surgical drainage, microbial growth within the bile culture was observed in a notable 82% of instances [9, 13, 26].

Nutritional status has been established as a factor correlated with infection control capability. In this context, it is noteworthy that a substantial proportion of patients displaying bactobilia were concurrently afflicted with hypoalbuminemia (72%). However, this association did not uniformly correlate with the occurrence of complications. Interestingly, colonization of the bile duct has been documented even among patients exhibiting favorable nutritional status (22%) [12, 36].

It is pertinent to acknowledge that certain variables, such as alcohol consumption and chronic pancreatitis, were not subjected to evaluation in the present study. It is imperative to emphasize that previous investigations, encompassing parameters like age, alcoholism, and cancer history, have not consistently revealed a definitive correlation with the occurrence of bactobilia [9, 12, 13, 26, 36].

The morbidity rate observed within the context of this study stood at 45%, a figure harmonizing with proportions presented by other researchers [9]. Moreover, this investigation facilitated the documentation of a comprehensive compilation of infectious-type complications, revealing their occurrence in 52% of patients exhibiting bactobilia. However, disparate findings have been reported across various publications. For instance, previous studies [9, 13] have reported statistically significant outcomes, indicating the presence of infections in 35% of patients displaying bactobilia, whereas Maatman et al. [27] recorded an infection rate of 13% (*p* > 0.05). Within the purview of the current study, statistically significant disparities emerged concerning the incidence of surgical site infections among patients with positive culture results (24%). This aligns with studies linking bactobilia to local infections (ranging from 17% to 23%) [20]. Nonetheless, it is important to acknowledge that certain studies have failed to consistently establish this association [9, 13, 21, 36].

Postoperative complications have traditionally been categorized based on the extent of resource utilization required for management. Historically, the Dindo-Clavien classification scale, as outlined in prior literature [21], has served as a framework. In this scale, a classification > II signifies the utilization of substantial resources or the necessity for reinterventions. Within the present study, major complications were documented in 17% of patients exhibiting bactobilia, a percentage that falls below the rates reported in other previous studies, where major complications have been documented to range as high as 54% [36]. This disparity could potentially be attributed to the cumulative experience gained within our hospital service, which may account for the discernible decrease in major complications. This, in turn, might elucidate the relatively infrequent occurrence of major complications within the cohort of patients demonstrating positive culture results.

As evident from the findings of the current study, *E. coli* has shown recurrent presence in positive bile cultures, demonstrating frequencies ranging from 34% to 37% [14, 15], which closely aligns with the reported occurrence here (37.8%). Moreover, similar occurrences to those detailed in the current investigation, involving Gram-negative microorganisms such as *Enterobacteriaceae*, as well as Gram-positive microorganisms including *Enterococcus*, have been previously documented [14, 15]. However, past microbiological investigations have indicated a heightened prevalence of *Enterococcus* species (52%) and a similar occurrence of *K. pneumoniae* (23%) [25]. It is important to highlight that local geographic differences in the prevalence of microorganisms have been recognized [12, 26, 27, 37].

The duration of hospitalization within the scope of this study remained unaffected by the results of the culture analysis. Nonetheless, upon comparison with other datasets, a lower rate of hospital stay was discernible, a phenomenon that may stem from variability in the design of each respective study [9].

In various investigations centered on complications associated with hepatobiliopancreatic surgery, mortality rates ranging from 1% to 5% have been reported [9, 27, 36, 38, 39]. These figures closely parallel the mortality rates encountered in the current study.

It is essential to acknowledge the inherent limitation of the retrospective nature of this study, which precludes the establishment of direct causality. Moreover, limitations of a retrospective cohort study include potential biases due to the reliance on historical data, difficulty, challenges in controlling for confounding variables, and the possibility of incomplete or inaccurate records impacting the validity of results. Additionally, the study design may not allow for the collection of certain types of data or control over the timing and quality of information recorded. While we acknowledge the importance of considering procedural variables such as surgical procedure, procedure duration, and preprocedural antibiotic use in the analysis of postoperative infections, these factors were not included in our study due to several limitations. Firstly, the procedures analyzed were all performed within the same pancreatobiliary area, limiting the variability in infection risk associated with different surgical procedures. Additionally, the protocolized nature of the procedures ensured consistent procedure durations and standardized preprocedural antibiotic use for all patients, reducing the variability in these factors across the study population. However, it is essential to recognize that infections can result from a combination of host factors and procedural factors. Future studies could explore the impact of procedural variables on infection outcomes in more detail, potentially by including a broader range of surgical procedures and assessing their association with infection risk. Furthermore, investigating the influence of procedure duration and preprocedural antibiotic use on postoperative infections could provide valuable insights into optimizing infection prevention strategies in surgical practice. Therefore, while our study provides valuable insights into the role of host factors in postoperative infections, it is essential to interpret the results within the context of these limitations and consider the potential influence of procedural variables in future research. On the other hand, the accrued experience has enabled the implementation of enhancements in the care protocol and informed decision-making. These elements have collectively contributed to the observed reduction in morbidity, mortality, and hospital stay among our patient cohort.

## 5. Conclusions

Bactobilia is a prevalent occurrence among patients afflicted with hepatobiliary and pancreatic disorders, thereby engendering an elevated risk of perioperative morbidity in surgical scenarios necessitating bile duct reconstruction. The act of obtaining intraoperative cultures is an uncomplicated and cost-effective procedure that bestows the capacity for timely and precisely directed interventions aimed at mitigating infectious complications. However, it is crucial to acknowledge that these conclusions should be interpreted considering the limitations of the study, including the exclusion of certain procedural variables and the focus on host factors.

## Figures and Tables

**Table 1:** General characteristics, cardiovascular risk factors, and preoperative factors.

Variable	Negative bile culture (*n* = 63)	Positive bile culture (*n* = 74)	*p* value
Age (mean)	59.2%	58.8%	0.152
Women	37 (58.7%)	35 (47.2%)	0.211
Bleeding > 400 ml	15 (23.8%)	17 (22.9%)	0.232
Preoperative biliary drainage	24(38.1%)	61(82.4%)	<0.001
Arterial hypertension	25 (39.7%)	30 (40.5%)	0.241
Diabetes mellitus	12 (19.1%)	7 (9.5%)	0.147
Albumin < 3.5 g/dl	14 (22.2%)	54 (72.9%)	<0.001
Prealbumin < 15	16 (25.4%)	21 (28.4%)	0.271
Intraoperative transfusion	10 (15.9%)	4 (5.4%)	0.109

**Table 2:** Surgical procedures.

Surgery	Negative bile culture (*n* = 63)	Positive bile culture (*n* = 74)	*p* value
Whipple	32 (50.8%)	34 (45.5%)	0.314
Biliary reconstruction	13 (20.6%)	28 (37.8%)	0.041
Hepatectomy	18 (28.6%)	12 (16.2%)	0.233

**Table 3:** Postoperative complications, duration of hospitalization, and mortality.

Variable	Negative bile culture (*n* = 63)	Positive bile culture (*n* = 74)	*p* value
One or more complications	23 (36.5%)	39 (52.7%)	0.172
Dindo‐Clavien > II	7 (11.1%)	13 (17.6%)	0.322
Infection	7 (11.1%)	26 (35.1%)	0.002
Operative site infection	5 (7.9%)	18 (24.3%)	0.010
Death < 30 days	3(4.8%)	6(8.1%)	0.311
Postoperative stay (days)	7 (5-10.5)^∗^	7 (5-11)^∗^	0.121

^∗^Data expressed in medians and their respective interquartile range.

**Table 4:** Details of positive bile cultures.

Parameter	%	*n*
*Escherichia coli*	37.8%	28
*Klebsiella pneumoniae*	21.6%	16
*Enterococcus*	12.2%	9
*Aeromonas*	9.5%	7
*Enterobacter*	6.8%	5
*Pseudomonas*	5.4%	4
*Hafnia*	2.7%	2
*Streptococcus*	1.4%	1
*Candida*	1.4%	1
*Citrobacter freundii*	1.4%	1

**Table 5:** Adjusted logistic regression model.

Variable	Beta	*p* value	OR	IC 95%
Positive culture	1.22	0.02	2.26	1.23-11
Bleeding > 400 ml	0.73	0.22	2.57	0.12-2.04
Diabetes mellitus	1.25	0.25	0,49	0.01-1.69
Age	0.005	0.8	3.84	0.96-1.04
Prealbumin < 15	1.18	0.72	2.32	0.44-3.59

## Data Availability

The datasets used and/or analyzed during the present study are available from the corresponding author upon reasonable request.
